# Hollow Gold-Silver Nanoshells Coated with Ultrathin SiO_2_ Shells for Plasmon-Enhanced Photocatalytic Applications

**DOI:** 10.3390/ma13214967

**Published:** 2020-11-04

**Authors:** Pannaree Srinoi, Maria D. Marquez, Tai-Chou Lee, T. Randall Lee

**Affiliations:** 1 Department of Chemistry and the Texas Center for Superconductivity, University of Houston, Houston, TX 77204-5003, USA; pannareeps@gmail.com (P.S.); mdmarqu2@gmail.com (M.D.M.); 2 Department of Chemical and Materials Engineering, National Central University, Jhongli City 32001, Taiwan; taichoulee@ncu.edu.tw

**Keywords:** nanoparticles, silica shells, metal nanoparticles, gold-silver nanoshells, core-shell nanoparticles

## Abstract

This article details the preparation of hollow gold-silver nanoshells (GS-NSs) coated with tunably thin silica shells for use in plasmon-enhanced photocatalytic applications. Hollow GS-NSs were synthesized via the galvanic replacement of silver nanoparticles. The localized surface plasmon resonance (LSPR) peaks of the GS-NSs were tuned over the range of visible light to near-infrared (NIR) wavelengths by adjusting the ratio of silver nanoparticles to gold salt solution to obtain three distinct types of GS-NSs with LSPR peaks centered near 500, 700, and 900 nm. Varying concentrations of (3-aminopropyl)trimethoxysilane and sodium silicate solution afforded silica shell coatings of controllable thicknesses on the GS-NS cores. For each type of GS-NS, scanning electron microscopy (SEM) and transmission electron microscopy (TEM) images verified our ability to grow thin silica shells having three different thicknesses of silica shell (~2, ~10, and ~15 nm) on the GS-NS cores. Additionally, energy-dispersive X-ray (EDX) spectra confirmed the successful coating of the GS-NSs with SiO_2_ shells having controlled thicknesses. Extinction spectra of the as-prepared nanoparticles indicated that the silica shell has a minimal effect on the LSPR peak of the gold-silver nanoshells.

## 1. Introduction

Harvesting solar energy and storing it in the form of a chemical, such as hydrogen, has garnered significant interest for renewable energy because it is the most abundant and free energy source available on earth [1,2]. Practical and sustainable approaches to solar energy include the utilization of photoelectrochemical reactions such as carbon dioxide reduction and water splitting to afford fuels (e.g., methane, methanol, and hydrogen) [3]. Of particular interest is the water-splitting reaction, for which the standard Gibbs free energy to produce hydrogen from water is greater than 237 kJ/mol and is equivalent to the wavelength of light in the range of 500–1100 nm [4]. Importantly, water is transparent and absorbs little or no light at those wavelengths. Therefore, various composites have been aggressively explored for use in water-splitting reactions due to their ability to absorb across the broad range of wavelengths in the solar spectrum [5].

Abundantly available metal oxide materials such as TiO_2_, ZnO, Al_2_O_3_, and Cu_2_O have been used as semiconductor photoelectrodes due to their photocatalytic properties [6,7]. However, most of the aforementioned metal oxide materials respond most efficiently to UV light, owing to their large bandgap (higher than 3.2 eV), while the bulk of solar radiation reaching the surface of the earth lies in the visible to near-infrared (NIR) regions [8]. Due to the limited range of absorption, there is a significant need to enhance the photocatalytic properties of metal oxides. Enhancement has been achieved by decorating the semiconductors with dyes, quantum dots, or noble metal/plasmonic nanoparticles [1,9].

Noble metal nanoparticles have been employed in various applications due to their unique chemical and physical properties that are typically different from the properties of the bulk material. One of the most interesting properties of noble metal nanoparticles, such as gold, silver, and copper, is the localized surface plasmon resonance (LSPR) [10,11]. Surface plasmon resonance occurs when the frequency of incident light matches the collective oscillation of surface electrons in the conduction band of the metal. Moreover, the LSPR extinction band can be tuned by varying the size, shape, morphology, and composition of the nanoparticles [12].

Coating a dielectric layer on metallic nanoparticles has the advantage of preventing bulk-metal behavior due to physical contact with neighboring particles or aggregation of unmodified metal nanoparticles. In other words, the dielectric shell plays an important role in enhancing the stability of metal nanoparticles. It is important to employ dielectric materials that are chemically inert, especially for plasmonic enhancement applications, because the optical properties of metal nanoparticles strongly depend on their size and shape. Furthermore, the aggregation of metal nanoparticles has a tremendous effect on the LSPR peak position, which leads to a change in the photo-responsive range of the materials. The SiO_2_ shell can maintain the original optical properties of the metal nanoparticles under high light intensity [13].

Additionally, the thickness of the SiO_2_ interlayer (i.e., the layer found between the noble metal nanoparticle and a photocatalytic material) has been reported to have an effect on enhancing the photocatalytic activity of photocatalysts [9,14]. The Li group reported the photocatalytic efficiency of an Ag@SiO_2_@TiO_2_ triplex core-shell photocatalyst as the thickness of the SiO_2_ interlayer was varied, and the authors found an increase in photocatalytic efficiency as the SiO_2_ thickness was decreased [9]. Unfortunately, the use of silver nanoparticles for these types of applications have the major drawback of their photo-responsive range being limited to only the visible region. As an alternative photoresponsive material, bimetallic core-shell nanoparticles (i.e., nanoshells) exhibit strong plasmon resonances shifted to longer wavelengths, compared to the plasmon resonance of the corresponding solid metal nanospheres, due to plasmonic coupling [15,16]. Specifically, gold-silver nanoshells (GS-NSs), due to their tunable LSPR peak in the range of visible to near-IR light, are good candidates to act as photosensitizers for photocatalysts. Furthermore, the Lee group also observed the benefits of having a silica interlayer between GS-NSs and a zinc indium sulfide (ZIS) outer layer when these composite particles were used as photocatalysts for hydrogen generation via water splitting [14]. Mechanistic studies showed that the silica interlayer blocks the transfer of hot electrons from the GS-NSs to ZIS [14]. Compared to GS-NSs with no SiO_2_ coating and those coated with 42 nm of SiO_2_, the use of a SiO_2_ coating 17 nm thick showed the greatest hydrogen production (2.6-fold increase compared to ZIS alone). The enhanced production observed for the sample with the thinnest SiO_2_ shell was attributed to a limited distance for the SPR-generated electromagnetic field to be effective, which translates to higher coupling between the ZIS photocatalyst and the GS-NS core. Therefore, a thinner SiO_2_ shell in the aforementioned type of photocatalyst can plausibly lead to plasmon-enhanced hydrogen evolution in water-splitting reactions.

A plethora of reports have been published on the synthesis of SiO_2_ shells on metal nanoparticles, with the most common route being modified Stöber methods by addition of tetraethoxysilane in ethanol solution [17]. Additionally, for aqueous media, Mulvany and co-workers have reported a method for the formation of thin silica shells [18]. Following this method, thicker SiO_2_ shells can be obtained by using the Stöber method after transferring the thin silica-coated particles into ethanol solution [18]. Furthermore, depending on the concentration of the silica precursors, the Stöber method can yield silica shells with a thickness in the range of 20 to 100 nm. Given the thickness ranges the aforementioned methods yield, there are only a limited number of literature examples that report highly reproducible methods to synthesize homogenous ultra-thin SiO_2_ shells on nanoparticle surfaces, especially on rough surfaces such as those on hollow gold-silver alloy nanoshells. As a result, we sought out to develop a reproducible method for the synthesis of homogenous ultra-thin SiO_2_ shells on GS-NSs. Herein, we describe the synthesis of hollow gold-silver nanoshells via the technique known as galvanic replacement [19] to give three distinct GS-NS samples having LSPR peaks centered at ~500, ~700, and ~900 nm. Each of the three types of synthesized GS-NSs were then coated with SiO_2_ having three different thicknesses (~2, ~10, and ~15 nm) by varying the concentration of (3-aminopropyl)trimethoxysilane (APTMS) and sodium silicate in the solution mixture.

## 2. Materials and Methods

### 2.1. Materials

All reagents were purchased from the indicated suppliers and used without further purification. Silver nitrate was purchased from Mallinckrodt (Staines-upon-Thames, United Kingdom). Trisodium citrate, potassium iodide (KI), ascorbic acid, (3-aminopropyl)trimethoxysilane (APTMS), and sodium silicate solution were purchased from Aldrich (St. Louis, MO, USA). Potassium carbonate was purchased from J. T. Baker (Phillipsburg, NJ, USA). Hydrogen tetrachloroaurate(III) hydrate was purchased from Strem (Newburyport, MA, USA). Water was purified to a resistivity of 18 MΩ.cm (Academic Milli-Q Water system; Millipore Corporation (Burlington, MA, USA)). All of the glassware used during the experiments were cleaned in a base bath, followed by aqua regia solution (3:1 HCl:HNO_3_), and then dried in the oven and cooled prior to use.

### 2.2. Preparation of Silver Nanoparticles Cores

Silver nanoparticles (Ag NPs) were prepared by modifying the KI-assisted ascorbic acid/citrate reduction protocol [20] and the method of Lee and Meisel [21]. Specifically, 1 mL of a 5 mM aqueous solution of ascorbic acid (AA) was added into 95 mL of boiling water, followed by boiling for an additional 1 min. An aliquot of AgNO_3_ (0.0167 g, 0.100 mmol) was dissolved in 2 mL of water, and then 2 mL of 1% trisodium citrate solution and 50 µL of 7 µM KI solution were added. The mixture was placed in an ultrasonic bath for 5 min and was injected into the boiling solution of ascorbic acid. The solution was brought to reflux for 1 h at 120 °C while stirring. The color of the solution quickly changed from colorless to yellow and turned yellowish green after 5 min of reaction, consistent with the presence of silver nanoparticles. The solution was allowed to cool to room temperature (rt) and centrifuged at 8000 rpm for 15 min. The nanoparticles were then redispersed in 12.5 mL water.

### 2.3. Preparation of Hollow Gold-Silver Nanoshells (GS-NSs)

The hollow GS-NSs were prepared by following an adaptation of a previously reported method [19,22] involving the use of a gold salt (K-Au) solution as the reducing agent. Specifically, 0.025 g of K_2_CO_3_ was added to 100 mL of water. After 5 min of vigorous stirring, 2 mL of 1% HAuCl_4_·H_2_O solution was injected. The color of the mixture changed from yellow to colorless after 30 min of reaction. The flask was covered with aluminum foil to shield it from light and was kept in the refrigerator overnight before being used. To deposit gold on the silver nanoparticles and etch their cores [19,22], various amounts of K-Au solution (10–100 mL) were added to 10 mL of silver nanoparticle solution under vigorous stirring. After 5 h, the solution was centrifuged at 7000 rpm for 15 min. The nanoshells were then redispersed in 10 mL of water at a concentration of 1.0 × 10^10^ particles/mL.

### 2.4. Preparation of Silica-Coated Hollow Gold-Silver Nanoshells

Silica-coated hollow GS-NSs were prepared by modification of a previously reported synthetic method [23]. Specifically, 2.5 mL of as-prepared GS-NS suspension was diluted to 40 mL and then mixed with 1 mL of 0.6–1.2 mM APTMS under the vigorous stirring. The mixture was heated to 80 °C, followed by the addition of 10–35 μL of sodium silicate solution. The solution was brought to reflux for 3 h under stirring. The solution was allowed to cool to rt and centrifuged at 6000 rpm for 20 min, and the supernatant decanted. The as-prepared nanoparticles were then redispersed in ethanol. This procedure was repeated three times to remove the residual small silica nanoparticles. Finally, the as-prepared nanoparticles were redispersed in 10 mL of ethanol for characterization and application.

### 2.5. Characterization of SiO_2_-Coated Gold-Silver Nanoshells

The size evaluation and imaging of the as-prepared nanoparticles were performed using a LEO-1525 scanning electron microscope (LEO Electron Microscopy Inc., NY, USA)(SEM) operating at an accelerating voltage of 1.5 kV. All samples were prepared by dropping diluted colloidal nanoparticles onto a silicon wafer and drying in an oven for 15 min. To confirm the size and morphology of nanoparticles at a high resolution, a JEM-2000 FX transmission electron microscope (JEOL, Tokyo, Japan)(TEM) operating at an accelerating voltage of 200 kV was used. All samples were deposited on 300 mesh holey carbon-coated copper grids and dried overnight before analysis. UV-Vis spectra were obtained using a Cary 50 scan UV-visible spectrometer (Agilent Technologies, CA, USA) over a wavelength of 200 to 1000 nm to measure the optical properties. The uncoated nanoparticles and SiO_2_-coated GS-NSs were suspended in water and ethanol, respectively, for the measurement. Energy-dispersive X-ray spectroscopy (EDX) data were collected by an EDX attached to the focused ion beam (FIB) instrument and by EDX attached to a JEOL-2200 transmission electron microscope (JEOL, Tokyo, Japan)(TEM) operating at an accelerating voltage of 200 kV. 

## 3. Results and Discussion

### 3.1. Synthesis of the Hollow Gold-Silver Nanoshells

Monodisperse silver nanoparticles having uniformly spherical shapes were synthesized by modifying a KI-assisted ascorbic acid/citrate reduction protocol. The ascorbic acid was used as the predominant reducing reagent of AgNO_3_, while KI was used to control the growth of Ag NPs via the adsorption on [111] facets [20]. Citrate has a minor role on the reduction of AgNO_3_ and is mainly used as a stabilizing agent on the newly formed Ag NPs [20]. Using the Ag NPs as starting materials, hollow gold-silver nanoshells were successfully synthesized via galvanic replacement [22]. As shown in Equation (1), Ag can be oxidized to Ag^+^, and the gold salts can be reduced to Au^0^ when the K-Au solution is added to the silver nanoparticle cores. This reaction occurs because the standard reduction potential of the Au^3+^/Au pair is higher than that of the Ag^+^/Ag pair, as shown in Equations (2) and (3).

3Ag + Au^3+^ → 3Ag^+^ + Au (1)

Ag^+^ + e^−^ → Ag   E^0^ = 0.80 V (2)

Au^3+^ + 3e^−^ → Au   E^0^ = 0.99 V (3)

**Scheme 1:**
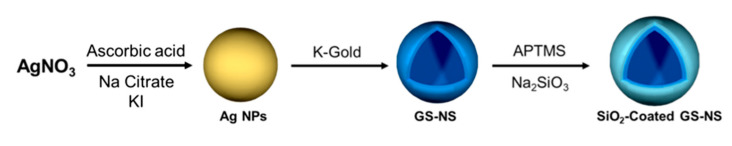
Synthesis of silica-coated hollow gold-silver nanoshells (GS-NS).

Silica-coated hollow gold-silver nanoshells were synthesized using a modification of a previously reported method to coat the GS-NSs with ~2, ~10, and ~15 nm silica shells [23]. As shown in Scheme 1, APTMS was used as a surface-directing agent, and sodium silicate (Na_2_SiO_3_) solution was used as the silica precursor to coat the GS-NSs. The NH_2_ groups of APTMS bind to the surface of the GS-NSs and the Si(OMe)_3_ groups are available for hydrolysis and condensation with Na_2_SiO_3_ to deposit a silica layer [23].

### 3.2. Morphology and Optical Properties of the Hollow Gold-Silver Nanoshells

**Figure 1:**
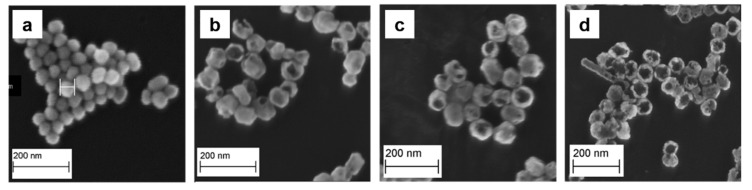
Scanning electron microscopy (SEM) images of (**a**) silver nanoparticles and gold-silver nanoshells synthesized by adding (**b**) 12.5 mL, (**c**) 30 mL, and (**d**) 150 mL of K-Au solution.

The morphologies of the GS-NSs were evaluated using SEM, as shown in Figure 1. Before performing the galvanic replacement with K-Au solution, we determined that the diameter of the silver nanoparticles was in the range of 50–60 nm (Figure 1a). Previous literature has established that addition of K-Au solution to the silver nanoparticles used for their reduction produces small pinholes, and that the pinholes became larger with increasing amounts of K-Au solution [22]. Regardless of the pinhole formation typically observed during the aforementioned process, the GS-NSs retained a diameter of 50–60 nm after the galvanic replacement reaction. The change observed in the morphology of the noble nanoparticles has an effect on their tunable optical properties, which leads to advantages in various kinds of optical applications, including enhanced photocatalysis in a specific region of light, and photothermal treatments and colorimetric sensors [24,25,26].

### 3.3. Optical Properties of the Hollow Gold-Silver Nanoshells 

**Figure 2:**
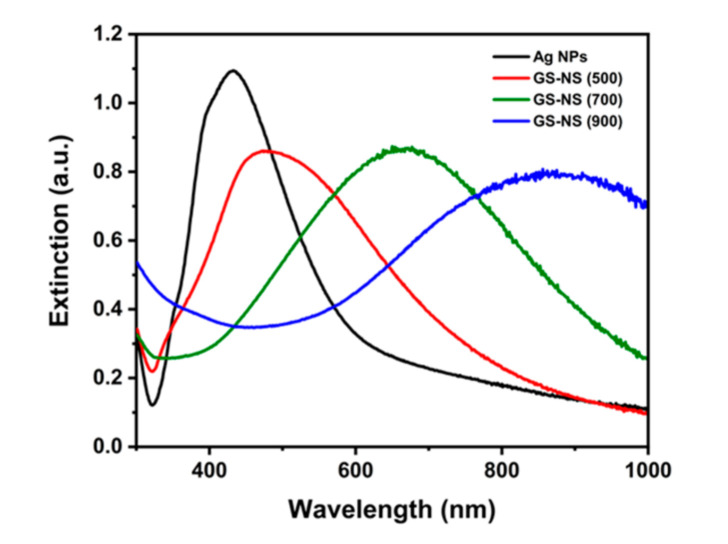
Extinction spectra of Ag NPs and gold-silver nanoshells having LSPR maxima at 500, 700, and 900 nm, denoted as GS-NS (500), GS-NS (700), and GS-NS (900). LSPR = localized surface plasmon resonance

Figure 2 illustrates the extinction spectra of silver nanoparticles and GS-NSs prepared using three different amounts of K-Au solution. The uncoated silver nanoparticles exhibit a sharp LSPR peak at ~430 nm. Upon increasing the amount of K-Au solution, the Au shell thickness of the GS-NSs decreases, and the LSPR peak shifts to a longer wavelength. Specifically, the GS-NSs synthesized with a 1:1 ratio of silver nanoparticles to K-Au solution showed an LSPR extinction peak at ~500 nm. Upon increasing the amount of K-Au solution to 1:3 and 1:15 of silver nanoparticles to K-Au solution, the GS-NSs exhibited LSPR extinction peaks at ~700 and ~900 nm, respectively. The LSPR peak is highly tunable in its position and is strongly dependent on the size of the nanoshell as well as shell thickness [22]. Furthermore, shifts in the LSPR position of the GS-NSs also depends on the composition of the nanoparticles. The amount of gold in the GS-NSs is proportional to the amount of K-Au solution added into the silver nanoparticles’ suspension. Therefore, adjusting the silver and gold ratios in the GS-NSs can be achieved by varying the amount of K-Au solution, which ultimately can be used to tune the LSPR of the GS-NSs into the visible light to near-IR regions.

### 3.4. Composition of the Hollow Gold-Silver Nanoshells

Additionally, TEM-EDX was used to identify the change of silver and gold atomic concentration in the as-prepared GS-NS nanoparticles. As summarized in Table S1 in the Supplementary Materials, the composition of GS-NS (500) showed a 5:1 ratio of Ag/Au. The Ag/Au ratio decreased when the LSPR peak position shifted to a longer wavelength. The Ag/Au ratios of GS-NS (700) and GS-NS (900) decreased to 2:1 and 1:1, respectively. The decrease of the Ag/Au ratio is consistent with the increase of K-Au solution to the silver nanoparticles suspension ratio used in the galvanic replacement reaction. As discussed in the previous section, Au^3+^ in the K-Au solution can be reduced to form Au^0^ on the nanoparticles, while the Ag atoms in original silver nanoparticle cores are dissolved in the solution as Ag^+^ and etched from the nanoparticles [22].

### 3.5. Morphology of the SiO_2_-Coated Gold-Silver Nanoshells

The GS-NSs synthesized with varying amounts of K-Au solution were each further coated with a silica shell. In these experiments, we observed that the silica shell thickness could be controlled by changing the concentration of APTMS and the sodium silicate solution, as shown in Table 1. Controllable silica shells with thicknesses of ~2, ~10, and ~ 15 nm were successfully coated on GS-NSs using this method. As mentioned above, APTMS acts as a surface-directing agent that binds to the surface of the GS-NSs, while the Si(OMe)_3_ tails react with the sodium silicate solution via hydrolysis reactions. Therefore, adjusting the concentration of the sodium silicate solution is an important parameter for controlling the thickness of the SiO_2_ layer on the surface of the GS-NSs. We note, however, that the concentration of APTMS is also an important factor for controlling the thickness of the SiO_2_ shell, as demonstrated by the thin SiO_2_ shell obtained from the GS-NS suspension when using low concentrations of APTMS (see Table 1).

**Figure 3:**
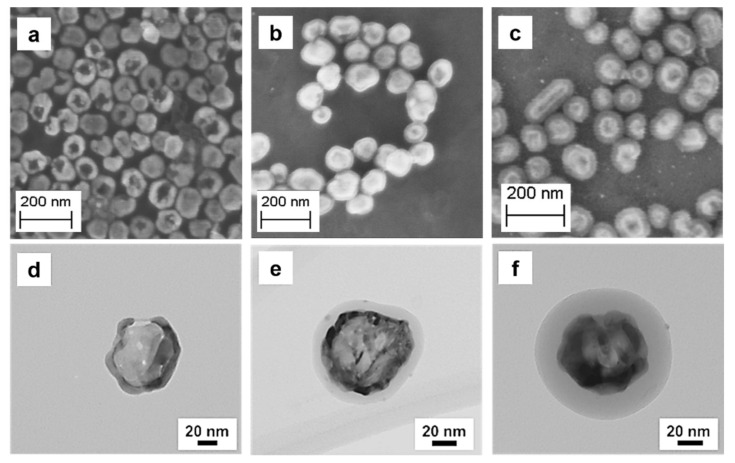
SEM and TEM images of silica-coated hollow gold-silver nanoshells with 900 nm LSPR extinction maxima. The thicknesses of the silica shell are (**a**,**d**) ~2 nm, (**b**,**e**) ~10 nm, and (**c**,**f**) ~15 nm.

Figure 3 shows SEM and TEM images of the SiO_2_-coated hollow GS-NS (900) having three different SiO_2_ thicknesses (~2, ~10, and ~15 nm). The SEM images indicate that the silica shell was homogenously coated on the surface of the individual GS-NSs. Importantly, the same procedure was applied to the GS-NSs with three different LSPR extinction peaks at 500, 700, and 900 nm to confirm the reproducibility of the synthesis method. As shown in Figure S1 in the Supplementary Materials, the GS-NSs with three different LSPR extinction peak positions (GS-NS (500), GS-NS (700), and GS-NS (900)) were successfully coated with the targeted silica thicknesses using this method. Moreover, as shown in the SEM images, coating with SiO_2_ shells caused no changes to the original morphologies of the hollow gold-silver nanoshells, which is consistent with the observed negligible effect on the optical properties of the GS-NSs (vide infra). The silica shell thickness distributions of the aforementioned nanoparticles were analyzed by using ImageJ version 1.53d. As summarized in Table 1, the average shell thickness of the SiO_2_-coated GS-NS (500) nanoparticles synthesized with three different conditions were 2.1 ± 1.0, 11.5 ± 1.7, and 15.8 ± 1.0 nm, respectively. In the case of the SiO_2_-coated GS-NS (700) nanoparticles, the silica shell thickness was controllable at 1.9 ± 1.0, 9.5 ± 0.9, and 16.0 ± 2.7 nm. Finally, the silica shell thicknesses of the SiO_2_-coated GS-NS (900) nanoparticles were 2.2 ± 1.3, 9.8 ± 0.8, and 15.1 ± 1.0 nm, respectively.

We note, however, that due to the limited resolution of the SEM images, the thicknesses of the silica shells are not clearly resolved in the SEM images. Consequently, we used TEM to confirm the thickness of the silica shells on the individual nanoparticles. As a representative example, Figure 3d–f show TEM images of SiO_2_-coated GS-NS (900) nanoparticles having different silica shell thicknesses. The thicknesses determined from the TEM images were in good agreement with the thicknesses determined from the SEM images (see Table 1 and Figure 3).

To provide further evidence of the silica shell on the GS-NSs following our controllable, reproducible method, TEM-EDX was used. The line spectra, shown in Figure S2 in the Supplementary Materials, confirms the presence of Si arising from the SiO_2_-shell, validating that silica shells can be grown with controllable thickness simply by changing the concentration of APTMS and sodium silicate in the solution. Supplementary Figure S2b shows the presence of gold, silver, and silicon in the line spectrum of SiO_2_-coated GS-NSs with a 2 nm SiO_2_ shell. The blue line of Si corresponding to the SiO_2_ shell shows ~2 nm Si atop the Ag and Au represented in green and red. The Si layer increases to ~10 to ~15 nm in the line spectra of SiO_2_-coated GS-NSs having ~10 and ~15 nm SiO_2_ shell thicknesses, respectively. Additionally, upon the increase of SiO_2_ thickness, the relative Si magnitude in the TEM line spectra of SiO_2_-coated GS-NSs having ~10 and ~15 nm shell thicknesses (Supplementary Figure S2d,f) is increased compared to Si in the SiO_2_-coated GS-NSs having 2 nm shell thicknesses. These results provide further confirmation that the thicknesses of the SiO_2_ shells can be precisely controlled using our method.

In a recent study, Lee et al. noted that a thin silica interlayer in a GS-NS@SiO_2_@ZIS nanocomposite enhanced hydrogen production efficiency [14]. The aforementioned nanocomposite was used as a photoelectrode in the photoelectrochemical water-splitting reaction for producing hydrogen gas [14]. As described above, the GS-NS@SiO_2_@ZIS nanocomposite with a 17 nm silica interlayer exhibited higher hydrogen production efficiency compared to the thicker silica shell (~42 nm) and a sample without a silica interlayer. Specifically, the GS-NS@SiO_2_@ZIS nanocomposites with a 17 nm SiO_2_ interlayer showed a hydrogen evolution rate of 0.131 ± 0.03 L/m^2^·h, while the GS-NS@SiO_2_@ZIS nanocomposites with a 42 nm SiO_2_ interlayer and those with no silica interlayer were approximately 0.07 and 0.09 L/m^2^·h, respectively. The SiO_2_-coated GS-NSs synthesized herein, shown in Figure 3, possess even thinner coatings than the SiO_2_ interlayers in the nanocomposites evaluated by Lee et al., making them prime candidates for photocatalytic hydrogen production.

### 3.6. Elemental Composition of the SiO_2_-Coated Gold-Silver Nanoshells

**Figure 4:**
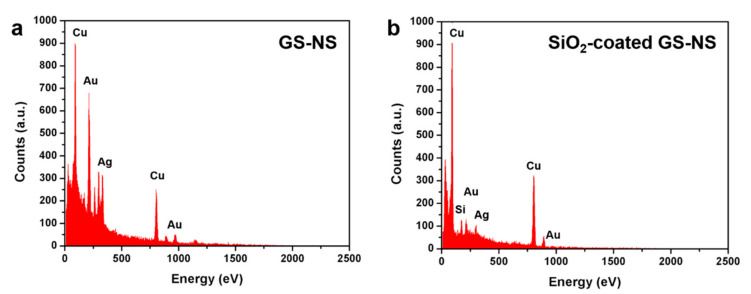
EDX spectra of (**a**) gold-silver nanoshells and (**b**) SiO_2_-coated gold-silver nanoshells having a 2 nm thick silica shell.

SEM-EDX data were collected to identify the elemental composition of the SiO_2_-coated GS-NSs. Figure 4 shows the presence of gold (Mα and Lα peaks at 212 and 971 eV, respectively), silver (Lα peak at 305 eV), and silicon (Kα peak at 174 eV) in the samples. The presence of copper peaks (Lα and Kα peaks at 93 and 894 eV, respectively) arise from the Cu tape used as the substrate in the SEM-EDX samples.

Furthermore, TEM-EDX was used to confirm the atomic concentration of silicon in the as-prepared SiO_2_-coated GS-NSs having varied thicknesses. As summarized in Table S2 in the Supplementary Material, the atomic percentage of silicon systematically increased across the series of samples. Specifically, for the SiO_2_-coated GS-NS (500) nanoparticles, the atomic percentage of Si increased from 15% for the ~2 nm thick sample to 41% and 71% for the ~10 and ~15 nm thick samples, respectively. The same trend was also observed for the SiO_2_-coated GS-NS (700) nanoparticles (i.e., 16%, 57%, and 66%) and the GS-NS (900) nanoparticles (i.e., 13%, 58%, and 75%). These results further confirm that SiO_2_-coated GS-NSs with ultrathin silica shells can be successfully synthesized by this method in a controllable manner.

### 3.7. Optical Properties of the SiO_2_-Coated Hollow Gold-Silver Nanoshells 

**Figure 5:**
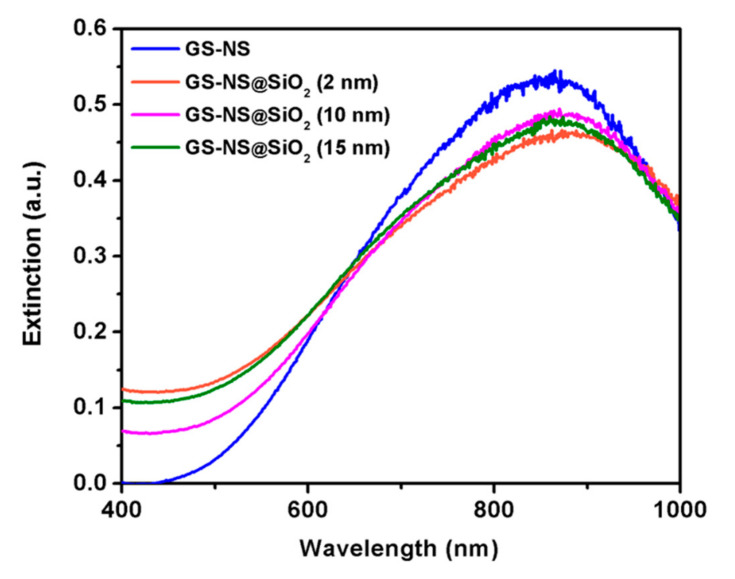
Extinction spectra of SiO_2_-coated GS-NS (900) with three different silica shell thicknesses.

Figure 5 shows the extinction spectra of the as-prepared nanoparticles with LSPR peak maximum at ~900 nm. Upon coating the GS-NSs with silica, the LSPR peak of the nanoparticles slightly shifted to a longer wavelength when compared to the uncoated GS-NSs. Specifically, the GS-NS (900) nanoparticles exhibited an LSPR extinction maximum at 850 nm, which shifted to 868, 870, and 873 nm for the SiO_2_-coated GS-NSs having ~2, ~10, and ~15 nm thick SiO_2_ shells, respectively. This small shift is likely due to a difference in the refractive index of SiO_2_ (1.49) as compared to water (1.33) [23]. We note also that the extinction maxima of the SiO_2_-coated GS-NSs having the three different silica thicknesses (~2, 10, and 15 nm) are essentially the same due to the broadness of the LSPR extinction peak (see Figure 5). This result demonstrates that the thickness of the silica shell has a minimal impact on the extinction spectra and thus the optical properties of the gold-silver nanoshells.

## 4. Conclusions

We have successfully synthesized hollow gold-silver nanoshells coated with silica shells of varying thicknesses by tuning the concentration of (3-aminopropyl)trimethoxysilane and sodium silicate solutions. Our strategy proceeded via the synthesis of silver nanoparticles (cores) in the size range of 60–80 nm using a modified KI-assisted citrate procedure followed by the formation of hollow gold-silver nanoshells via galvanic replacement. Extinction spectra of the GS-NSs demonstrated that these particles are tunable in the visible to NIR region (500–900 nm) by varying the ratio of silver nanoparticles to K-Au solution. Analyses by SEM and TEM were used to establish the size and morphology of the as-prepared nanoparticles and the thickness of the silica coating. The TEM images showed that the silica shell can be obtained at thicknesses of 2, 10, and 15 nm atop the GS-NSs following our method. Importantly, we note that neither of the three obtained SiO_2_ shells had detrimental effects on the localized surface plasmon resonance (LSPR) peak of the gold-silver nanoshells. On the whole, the studies described herein demonstrated that SiO_2_-coated GS-NSs having ultrathin silica shells are promising materials for plasmon-enhanced photocatalytic applications [9,14].

## Figures and Tables

**Table 1:** Concentration and volume of colloidal GS-NS suspension and silica precursors used to obtain silica shells of varying thicknesses.

Nanoparticles	APTMS		Na_2_SiO_3_	Silica Shell Thickness (nm)
	Concentration (µM)	Volume (mL)	Volume (µL)	
2.5 mL GS-NS (500)	0.6	1.0	10	2.1 ± 1.0
	1.0	1.0	25	11.5 ± 1.7
	1.5	1.0	35	15.8 ± 1.0
2.5 mL GS-NS (700)	0.6	1.0	10	1.9 ± 1.0
	1.0	1.0	25	9.5 ± 0.9
	1.5	1.0	35	16.0 ± 2.7
2.5 mL GS-NS (900)	0.6	1.0	10	2.2 ± 1.3
	1.0	1.0	25	9.8 ± 0.8
	1.5	1.0	35	15.1 ± 1.0

## Supplementary Materials

The following are available online at https://www.mdpi.com/1996-1944/13/21/4967/s1, Table S1: EDX-Derived Composition of GS-NSs with Different LSPR Extinction Peaks, Figure S1: SEM images of silica-coated GS-NSs with different LSPR peak positions. (a–c) GS-NS (500), (d–f) GS-NS (700), and (g–i) GS-NS (900) with ~2, ~10, and ~15 nm, respectively, Figure S2: STEM images and corresponding EDX line scan spectra of SiO_2_-coated GS-NSs with (a, b) 2 nm, (c, d) 10 nm, and (e, f) 15 nm silica shell, Table S2: EDX-Derived Composition of the SiO_2_-Coated GS-NSs.
