# Supplementary material for: Hollow Gold-Silver Nanoshells Coated with Ultrathin SiO2 Shells for Plasmon-Enhanced Photocatalytic Applications
Source: Materials (Basel). 2020 Nov 4;13(21):4967. doi: 10.3390/ma13214967 (PMC7672541; doi:10.3390/ma13214967)
Supplement: Supplementary file 1 [file materials-13-04967-s001.pdf]

materials

Supplementary Materials

Hollow Gold-Silver Nanoshells Coated with Ultrathin SiO_2 Shells for Plasmon-Enhanced Photocatalytic Applications

Pannaree Srinoi,[1] Maria D. Marquez,[1] Tai-Chou Lee,[2] and T. Randall Lee[1,]*

[1]Department of Chemistry and the Texas Center for Superconductivity, University of Houston, Houston, TX 77204-5003, USA

[2]Department of Chemical and Materials Engineering, National Central University, Jhongli City 32001, Taiwan

* Correspondence:trlee@uh.edu

Table S1. EDX-Derived Composition of Au-Ag Nanoshells with Different LSPR[1] Extinction Peaks.

Nanoparticles	Atomic Concentration (%)		
	Ag	Au	Ag/Au ratio
GS-NS (500)	84	16	5
GS-NS (700)	63	37	2
GS-NS (900)	54	45	1

[1]LSPR = localized surface plasmon resonance

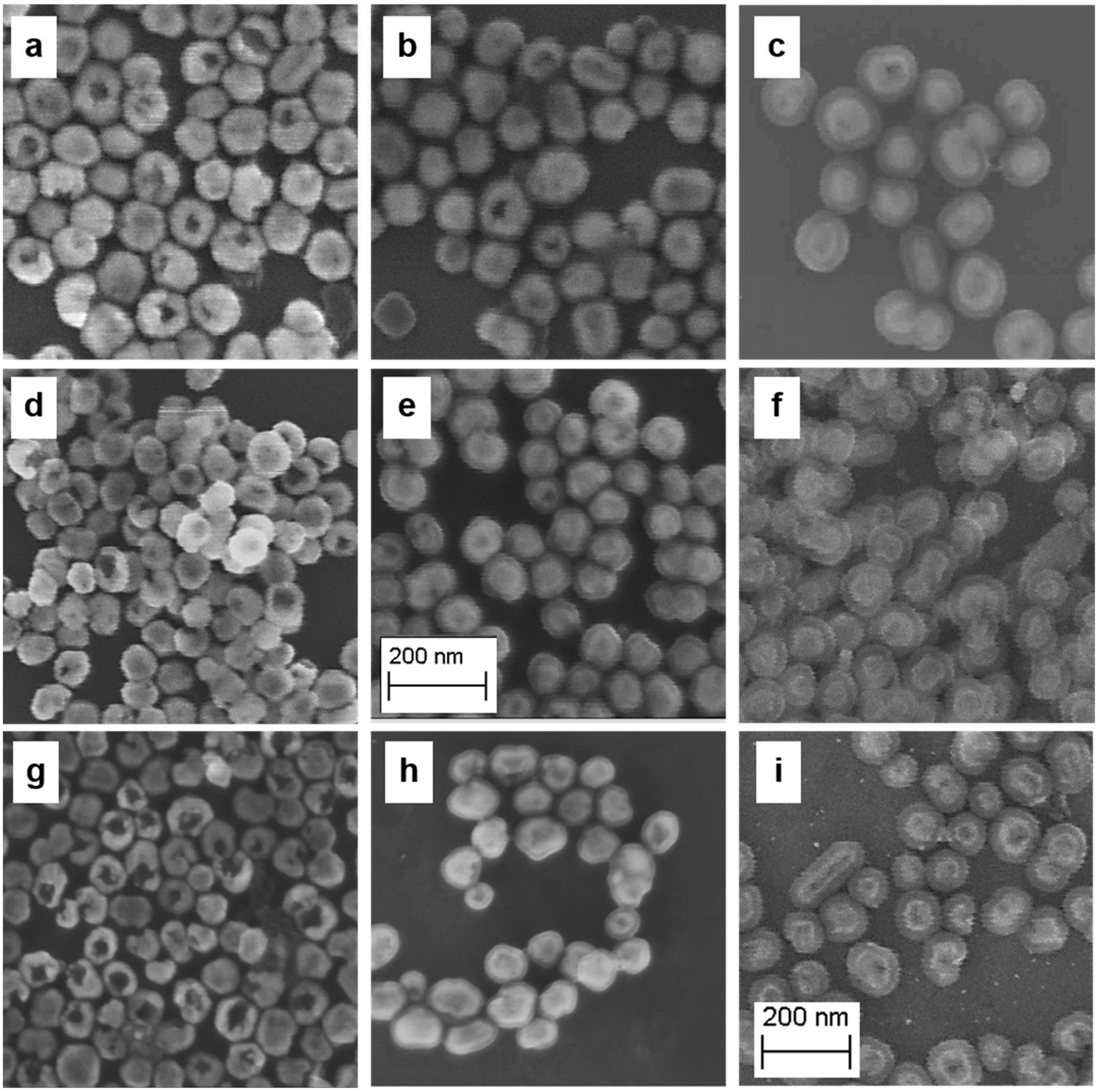

Figure S1. SEM images of silica-coated gold-silver nanoshells with the indicated LSPR peak positions. (**a–c**) GS-NS (500), (**d–f**) GS-NS (700), and (**g–i**) GS-NS (900) with ~2 nm, ~10 nm, and ~15 nm, respectively. LSPR = localized surface plasmon resonance

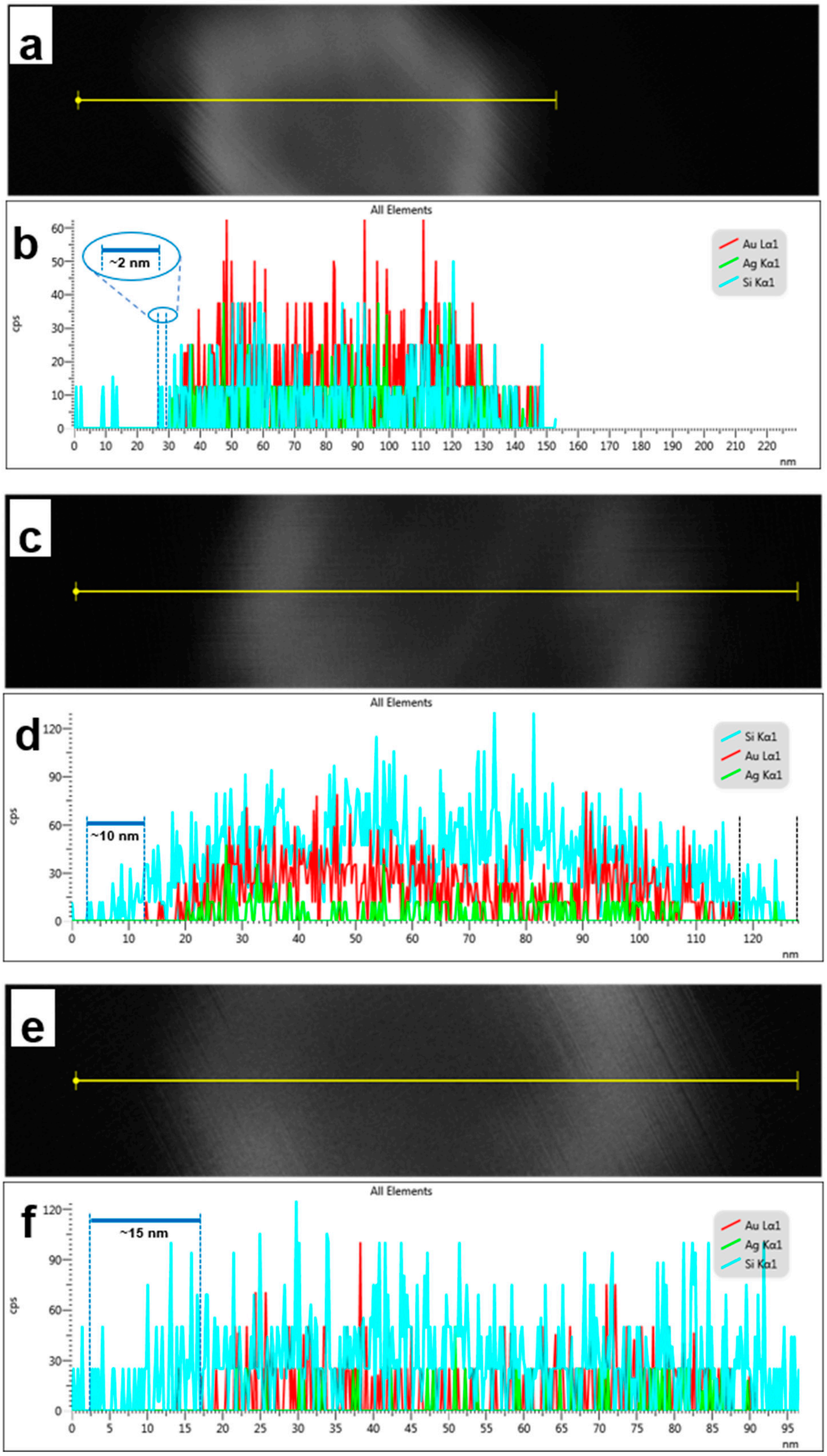

Figure S2. STEM images and corresponding energy-dispersive X-ray (EDX) line scan spectra of SiO_2-coated gold-silver nanoshells with (**a**,**b**) 2 nm, (**c**,**d**) 10 nm, and (**e**,**f**) 15 nm silica shell.

Table S2. EDX-Derived Composition of the SiO_2-Coated Gold-Silver Nanoshells.

Nanoparticles	SiO_2 Thickness (nm)	Atomic Concentration (%)		
		Ag	Au	Si
GS-NS (500)	2	75	15	15
	10	49	9	41
	15	24	5	71
GS-NS (700)	2	58	26	16
	10	28	16	57
	15	21	13	66
GS-NS (900)	2	43	44	13
	10	24	18	58
	15	14	11	75

© 2020 by the authors. Licensee MDPI, Basel, Switzerland. This article is an open access article distributed under the terms and conditions of the Creative Commons Attribution (CC BY) license (http://creativecommons.org/licenses/by/4.0/).
